# Expression of *STAT5*, *COX-2* and *PIAS3* in Correlation with NSCLC Histhopathological Features

**DOI:** 10.1371/journal.pone.0104265

**Published:** 2014-08-19

**Authors:** Dorota Pastuszak-Lewandoska, Daria Domańska, Karolina H. Czarnecka, Jacek Kordiak, Monika Migdalska-Sęk, Ewa Nawrot, Justyna Kiszałkiewicz, Adam Antczak, Paweł Górski, Ewa Brzeziańska

**Affiliations:** 1 Department of Molecular Bases of Medicine, Medical University of Lodz, Lodz, Poland; 2 Department of Chest Surgery, General and Oncological Surgery, University Hospital No. 2, Medical University of Lodz, Lodz, Poland; 3 Department of General and Oncological Pulmonology, Medical University of Lodz, Lodz, Poland; 4 Department of Pneumology and Allergology, Medical University of Lodz, Lodz, Poland; Virginia Commonwealth University, United States of America

## Abstract

Signal transducers and activators of transcription (STATs), their inhibitors and cyclooxygenase-2 (COX-2) participate in transformations of many various types of cancers. The aim of the present study was to evaluate the relationship between *STAT5A/B*, *COX-2*, and *PIAS3* mRNA expression and tumor staging, metastasis status, and histopathological subtype in 71 patients with confirmed non-small cell lung cancer (NSCLC) diagnosis. Total RNA was isolated from NSCLC tissue samples and the expression of the studied genes was assessed using TaqMan probes in real-time PCR assay. The expression levels of *STAT5A*, *STAT5B*, and *COX-2* genes were increased in 69%, 79%, and 71% NSCLC samples respectively, while *PIAS3* expression was decreased in the majority (69%) of the studied tissues. Statistically significant differences were observed between *STAT5* isoforms (P = 0.0008), with higher expression of *STAT5B*. We found statistically significant positive correlation between *STAT5B* and *COX-2* (rho = 0.045), and significant negative correlation between *STAT5B* and *PIAS3* (rho = −0.049). The negative correlation between *STAT5B* and *PIAS3* (rho = −0.43) was also observed in T2a+T2b tumor group. Additionally, *STAT5B* and *COX-2* expression levels were significantly different between T1a+T1b and T2a+T2b tumors (P = 0.002 and P = 0.041, respectively), with higher expression of both genes in T2 tumor stage. *PIAS3* expression was significantly lower in NSCC subtype as compared with SCC subtype (P = 0.017). Also, STAT5A and STAT5B immunoexpression was assessed, and the results indicated significantly higher protein levels in NSCLC patients as compared with controls (P = 0.048 and P = 0.034, respectively). High STAT5B immunoexpression was positively correlated with *STAT5B* gene expression in tumors (rho = 0.755). STAT5B protein level was also significantly higher in T2a+T2b tumors, reflecting high *STAT5B* gene expression in this group. There was no statistically significant association between mRNA and protein expression levels of the studied genes and patients' characteristics: age, gender, smoking. The obtained results highlight the importance of the genes *STAT5B* and *COX-2* in lung cancer progression.

## Introduction

It is documented that despite of more and more modern treatment options, lung cancer is one of the leading reason of cancer related deaths in the world. Non-small cell lung cancer (NSCLC) is recognized as the most common – accounting for 75–85% – among all lung malignant tumors [1]. With advances in molecular biology, the detection and analysis of changes in expression levels of many important genes involved in signaling pathways may supply predictive molecular markers harboring diagnostic and/or prognostic value for NSCLC.

In many human cancers, including NSCLC, one of the key pathways that promote cellular survival or cell growth is Janus kinase/signal transducers and activators of transcription (JAK/STAT) pathway. It is one of the pleiotropic cascades of molecules involved in signal transduction for proliferation, development and apoptosis [2], [3]. STAT (signal transducers and activators of transcription) protein family of transcription factors consists of seven members: 1–4, 5A, 5B, and 6 [2]. Among them, and beside STAT3, the oncogenic activity of STAT5 was documented both *in vitro* and *in vivo* [4], [5]. After phosphorylation, the two STAT5 proteins, STAT5A and STAT5B, as homo- or heterodimer, may translocate to the nucleus and bind to specific STAT5 response elements of target gene promoters, thus influencing their expression [6]. It is recognized that *STAT5A* and *STAT5B* isoforms are encoded by two tandemly linked genes on chromosome 17q11.2 [7]. They act as independent transcription factors [8] and modulate important cellular processes in different manner in normal and malignant cells [9]. Generally, active STAT5 promotes cell cycle progression, proliferation, invasion, angiogenesis, and inhibits apoptosis.

The overexpression of STAT5 has been recognized in several types of human tumors, mainly in breast and prostate cancers [10]–[13]. However, as so far, the data on the role of STAT5 in NSCLC cells, as well as on its activation status in NSCLC is still very limited. Recently, it has been documented that besides several cytokines, hormones and growth factors, EGF influences the *STAT5* expression in human lung adenocarcinoma cell line thus leading to the increased cyclooxygenase-2 (COX-2) expression [14]. COX-2, belonging to the COX enzyme family (COX-1, COX-2 and COX-3) [15], is a key enzyme in the biosynthesis of prostaglandins (PG). COX-2 is involved in the initiation and progress of tumors *in situ* and its overexpression is frequently recognized in many tumor types, including NSCLC [16]–[18]. However, carcinogenic effect of COX-2 upregulation in relation to the expression level of *STAT5* in NSCLC patients has not been investigated yet.

On the other hand, as a modulator of activity of STAT5, a protein inhibitor of activated STAT3 (PIAS3), which regulates different DNA binding transcription factors implicated in the immune response (e.g., NFκB, SMAD, and MITF), was recognized in breast cancer [19]. To date, only a small number of reports focused on PIAS3 participation in cancers involving lung tumors, has been published [20]–[23]. Additionally, although it is known that PIAS3 plays a vital role in oncogenic process influencing STAT3 protein, interaction between PIAS3 and STAT5 has not been fully recognized yet. The aim of our study was to determine the relationship between *STAT5*, *PIAS3* and also *COX-2* and their reciprocal relationship on transcriptional level in NSCLC patients. To achieve this goal, we assessed the mRNA expression of these genes and their association with histopathological features of NSCLC tumors as well as clinical characteristic of patients. The prespecified hypothesis tested was that *STAT5*, *COX-2* and *PIAS3* expression levels were modified in non-small cell lung cancer, playing a role in lung carcinogenesis. Additionally, we analyzed the levels of STAT5A and STAT5B proteins in the studied samples.

## Materials and Methods

The study has been approved by the Ethical Committee of the Medical University of Lodz, Poland no. RNN/64/11/KE. Written informed consent was obtained from each patient.

### 1. Characterization of the NSCLC tissue samples and patients clinical characteristics

Biological material (lung tissue) was obtained from 84 patients admitted to the Department of Thoracic Surgery, General and Oncologic Surgery, Medical University of Lodz, Poland, between July 2010–June 2012. Based on the results of preoperative cytological/histological assessment, the patients were qualified for surgery and were treated by either lobectomy or pneumonectomy.

Immediately after resection, lung tissue samples (100–150 mg) and the adjacent non-cancerous macroscopically unchanged tissues (100 mg; 10 cm distant from the primary lesion) obtained from the same patients were placed in a stabilization buffer RNAlater®. Each tissue sample was divided into smaller parts (30–50 mg) for individual analysis. All samples were frozen at −80°C.

The resected lesions were post-operatively histhopathologically evaluated and classified according to the AJCC staging as well as TNM classification (pTNM). Based on the results, from the group of 84 patients, 12 of them were excluded due to a concomitant malignancy and the suspected metastasis to the lung, in the case of one patient, there was no full clinical information. The final study group included seventy one (n = 71) patients with confirmed NSCLC diagnoses. Histopathological assessments of tumor specimens were obtained from pathomorphological reports, and were as follows: squamous cell carcinoma (SCC, n = 41), non-squamous cell carcinoma (NSCC, n = 30). Histopathological verifications of non-small cell lung carcinoma tissues are shown in **Table 1**. The studied group consisted of 25 women, mean age 63±8.717 and 46 men, mean age 65±8.234. All cases were primary tumors without chemo- or radiotherapy treatment. The smoking history was available for all patients: 5 patients were non-smokers, and 66 were smokers or former smokers. They were divided into groups according to their smoking habits: time of tobacco addiction and amount of cigarettes smoked – the latter was presented as Pack Years (PYs) and was calculated according to the NCI Dictionary of Cancer Terms (1 Pack Year is equal to 20 cigarettes smoked per day for 1 year), see **Table 2**.

**Table 1:** Histopathological verifications of NSCLC tissue samples.

**Histopathological type of NSCLC**	squamous cell carcinoma (SCC)	41 (57.75%)
	non-squamous cell carcinoma (NSCC)	30 (42.25%)
**AJCC** *	AJCC IA	14 (19.72%)
	AJCC IB	11 (15.49%)
	AJCC IIA	13 (18.31%)
	AJCC IIB	10 (14.08%)
	AJCC IIIA/IIIB	23 (32.39%)
**pTNM** *	T1a+T1b	19 (26.76%)
	T2a+T2b	33 (46.48%)
	T3+T4	19 (26.76%)

*AJCC – American Joint Committee on Cancer Staging according to the IASCLC Staging Project 7th ed. (2010) Cancer.

*pTNM — post-operative Tumor Node Metastasis classification according to the WHO Histological Typing of Lung Tumor.

**Table 2:** Characteristics of NSCLC patients in terms of tobacco addiction and consumption (the amount of cigarettes smoked per day and PYs).

Tobacco addiction and consumption		n = 71
**Smokers**		66 (92.96%)
**Non-smokers**		5 (7.04%)
**The smoking period**	<40 years	37 (52.11%)
	≥40 years	29 (40.85%)
**The amount of cigarettes smoked**	10–15 cigarettes per day	6 (9.09%)
	20 cigarettes per day (1 pack)	43 (65.15%)
	30–40 cigarettes per day (1.5–2 packs)	17 (25.76%)
**Pack Years (PYs)**	up to 40 PYs	30 (45.45%)
	≥40 PYs	36 (54.54%)

### 2. RNA extraction, real-time PCR

Total RNA was extracted from lung tissues (cancer tissue obtained from the center of lung lesion and macroscopically unchanged lung tissue obtained from the most distant site from the resected lesion) using Universal RNA Purification Kit (Eurix, Poland), and according to the manufacturer's recommendations. The quality and quantity assessments of RNA samples were determined by minielectrophoreses in polyacrylamide gel (Agilent 2100 Bioanalyzer, Agilent, USA), using RNA 6000 Pico/Nano LabChip kit (Agilent Technologies, USA). Complementary DNA (cDNA) was transcribed from 100 ng of total RNA, using a High-Capacity cDNA Reverse Transcription Kit (Applied Biosystems, USA) in a total volume of 20 µl per reaction. Reverse transcription (RT) master mix contained: 10× RT buffer, 25× dNTP Mix (100 mM), 10× RT Random Primers, MultiScribe™ Reverse Transcriptase, RNase Inhibitor and nuclease-free water. RT reaction was performed in a Personal Thermocycler (Eppendorf, Germany) in the following conditions: 10 minutes at 25°C, followed by 120 minutes at 37°C, then the samples were heated to 85°C for 5 seconds, and hold at 4°C. The relative expression was assessed using TaqMan probes: Hs00234181_m1, Hs00273500_m1, Hs00153133_m1, Hs00180666_m1 for the studied genes *STAT5A*, *STAT5B*, *COX-2* and *PIAS3*, respectively, as well as for *β-actin* (ACTB, Hs99999903_m1), as the reference gene. The PCR mixture contained: cDNA (1 to 100 ng), 20× TaqMan® Gene Expression Assay, 2× TaqMan® Gene Expression Master Mix, RNase-free water in a total volume of 20 µl. The qPCR reactions were performed in Applied Biosystems 7900HT Fast Real-Time PCR System for 39 cycles, with annealing temperature of 60°C, repeated 3 times for each sample. The relative expression of the studied samples were assessed using the Comparative delta-delta C_T_ method (TaqMan Relative Quantification Assay software) and presented as RQ value, adjusted to *β-actin* expression level. Macroscopically unchanged lung tissue served as calibrator sample.

### 3. Tissue homogenization, ELISA assays

Lung tissue samples (30–50 mg) were rinsed in ice-cold PBS buffer (0.01 mol/l, pH 7.0–7.2) and homogenized in 5 ml of PBS. The resulting suspension was subjected to two cycles of freezing and thawing. Then, the homogenates were centrifuged for 5 minutes at 5000×g, the supernatant was removed and the suspension was aliquoted and stored at −80°C until further analysis.

STAT5A and STAT5B immunoexpression levels in lung tissue homogenates were assessed using ELISA Kit for Signal Transducer And Activator Of Transcription 5A (SEB738Hu, Uscn Life Science Inc., China) and ELISA Kit for Signal Transducer And Activator Of Transcription 5B (SEB727Hu, Uscn Life Science Inc., China), according to the manufacturer's procedure. The intensity of the final colorimetric reaction, in proportion to the amount of protein bound, was measured in a plate reader (ELx800, BioTek) at 450 nm. The obtained results were compared to the standard solution of known concentrations (0.312–20 ng/ml).

### 4. Statistical analysis

ANOVA Kruskal-Wallis' test and U Mann-Whitney test, were used to compare the levels of relative expression values (RQs) of *STAT5A*, *STAT5B*, *COX-2* and *PIAS3* among NSCLC subtypes (SCC, NSCC).

The Spearman's rank correlation coefficient, U Mann-Whitney's test and ANOVA Kruskal-Wallis' test were performed in order to evaluate the relationship between the expression levels (RQ values) of the studied genes (*STAT5A*, *STAT5B*, *COX-2* and *PIAS3*), between RQs and the examined parameters (patient characteristics: age, gender and tumor staging according to pTNM and AJCC classification), as well as to find correlations regarding STAT5A and STAT5B protein levels in the studied samples. The accepted level of statistical significance was estimated at P<0.05. The results of relative expression levels of the studied genes are presented as RQ (relative quantification) means ± SEM and RQ means ± SD values. Statistica for Windows 10.0 program was applied for calculations.

## Results

### 1. Relative expression levels of *STAT5A*, *STAT5B*, *COX-2* and *PIAS3*

Relative expression levels of the studied genes (*STAT5A, STAT5B, COX-2, PIAS3*), expressed as RQ values, were determined using delta-delta C_T_ method, adjusted to the expression of *β-actin* (endogenous control) and relative to the expression level of calibrator (macroscopically unchanged lung tissue) for which RQ = 1.

The obtained RQ values for the individual genes were correlated with histopathological NSCLC subtypes (SCC, NSCC), tumor staging (pTNM, AJCC), patients' age, gender and smoking history.

*STAT5A* expression was increased (RQ value>1) in the majority (69%) of the studied samples. Regarding the individual histotypes, it was increased in a greater proportion in SCC group, but there were no statistically significant differences. *STAT5B* expression was increased in all studied histopathological NSCLC subtypes, in a range of 70%–85%, depending on a histotype. Expression levels of *STAT5* isoforms (*STAT5A* and *STAT5B*) were statistically significant different (P = 0.0008; Spearman's rank correlation coefficient), with higher expression level displayed by *STAT5B*. The expression level of *COX-2* was nearly uniformly increased in all NSCLC subtypes, at the level of 70%. In contrast, *PIAS3* expression was decreased (RQ value<1) and it was observed in all studied histopathological NSCLC subtypes, in a range of 66%–73%, depending on a histotype. Statistically significant differences were found between SCC and NSCC subtypes (P = 0.017; U Mann-Whitney's test), with significantly lower gene expression observed in NSCC samples. The results regarding genes' expression levels are summarized in **Table 3**.

**Table 3:** The expression levels (RQ values) of the studied genes in individual histopathological NSCLC subtypes.

Gene	Histopathological NSCLC subtype	Mean RQ value (range)	Number (percentage) of samples with	
			RQ value>1	RQ value<1
***STAT5A***	SCC (n = 41)	3.25 (0.25–4.49)	31 (75.60%)	10 (24.39%)
	NSCC (n = 30)	2.155 (0.05–4.54)	18 (60.00%)	12 (40.00%)
	**Total (n = 71)**	**2.75 (0.05–4.54)**	**49 (69.01%)**	**22 (30.99%)**
***STAT5B***	SCC (n = 41)	8.92 (0.67–59.29)	35 (85.37%)	6 (14.63%)
	NSCC (n = 30)	9.26 (0.77–864.17)	21 (70.00%)	9 (30.00%)
	**Total (n = 71)**	**9.13 (0.77–864.17)**	**56 (78.87%)**	**15 (21.13%)**
***COX-2***	SCC (n = 41)	9.34 (0.10–58.21)	29 (70.73%)	12 (29.27%)
	NSCC (n = 30)	4.89 (0.03–97.53)	21 (70.00%)	9 (30.00%)
	**Total (n = 71)**	**3.17 (0.24–20.89)**	**50 (70.42%)**	**21 (29.58%)**
***PIAS3***	SCC (n = 41)	0.21 (0.16–1.74)	14 (34.15%)	27 (65.85%)
	NSCC (n = 30)	0.94 (0.02–1.12)	8 (26.67%)	22 (73.33%)
	**Total (n = 71)**	**0.54 (0.02–14.91)**	**22 (30.99%)**	**49 (69.02%)**

### 2. Statistical analysis of relationship between studied gene expression levels and clinical features of patients and tumor characteristics

There was no statistically significant correlation between RQ values of *STAT5A* and the clinical features of NSCLC patients, i.e., patients' age (P = 0.48), gender (P = 0.54), and history of smoking assessed as PY (P = 0.87), as well as histopathological features of tumor, i.e., pTNM classification (P = 0.34), AJCC classification (P = 0.83), NSCLC subtypes (SCC *vs* NSCC, P = 0.62) (ANOVA Kruskal-Wallis test, U Mann-Whitney's test followed by Spearman's rank correlation coefficient).

**Figure 1:**
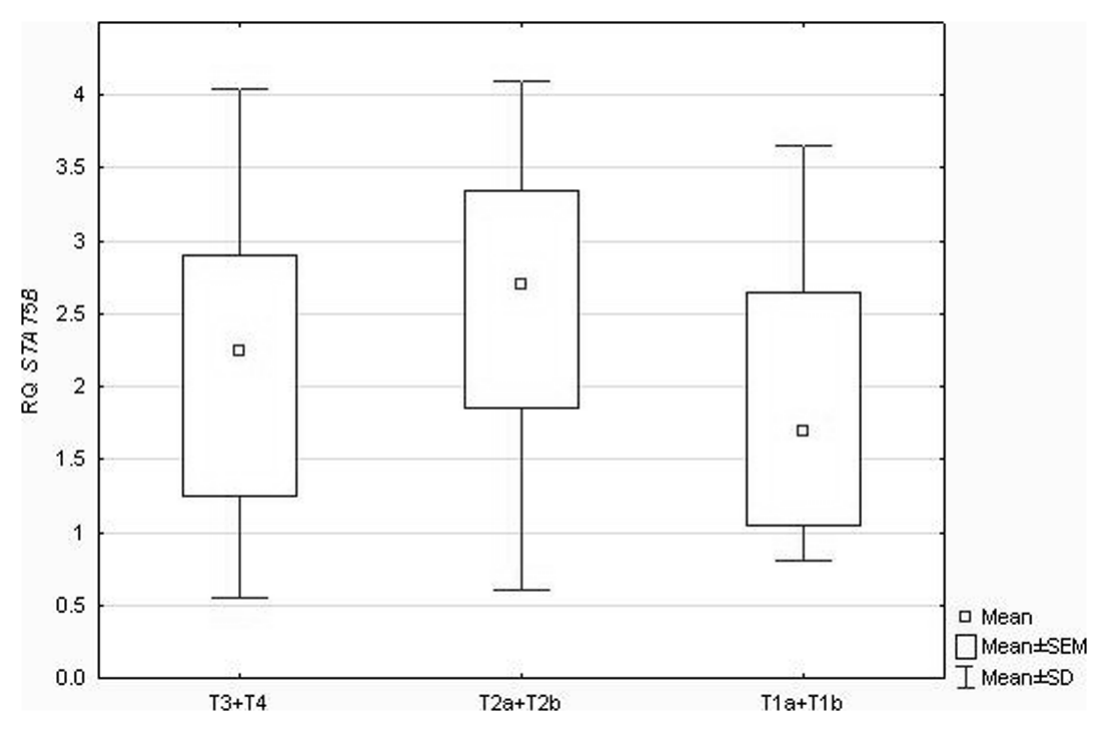
Box-and-whisker plots, representing *STAT5B* expression levels (mean RQ values) in the studied tumor groups classified according to pTNM staging in relation to their size.

Similarly, statistical analysis concerning *STAT5B* did not reveal significant correlations between its expression levels and patients' age (P = 0.59), gender (P = 0.45), history of smoking (P = 0.66), NCSLC subtypes (P = 0.85) and tumor AJCC staging (P = 0.23) (ANOVA Kruskal-Wallis test, U Mann-Whitney's test followed by Spearman's rank correlation coefficient). However, statistically significant differences were found in relation to tumor size classified according to pTNM staging (P = 0.034; ANOVA Kruskal–Wallis test), as presented in **Figure 1**. Neuman-Keuls' multiple comparison test revealed statistically significant differences between T1a+T1b *vs* T2a+T2b group (see **Table 4**).

**Table 4:** The results of Neuman-Keuls' multiple comparison test regarding differences in *STAT5B* RQ values in the studied tumor groups classified according to pTNM staging in relation to the tumor size.

	T1a+T1b	T2a+T2b	T3+T4
**T1a+T1b**		**0.001782**	1000000
**T2a+T2b**	**0.001782**		0.723456
**T3+T4**	1.000000	0.723456	

**Figure 2:**
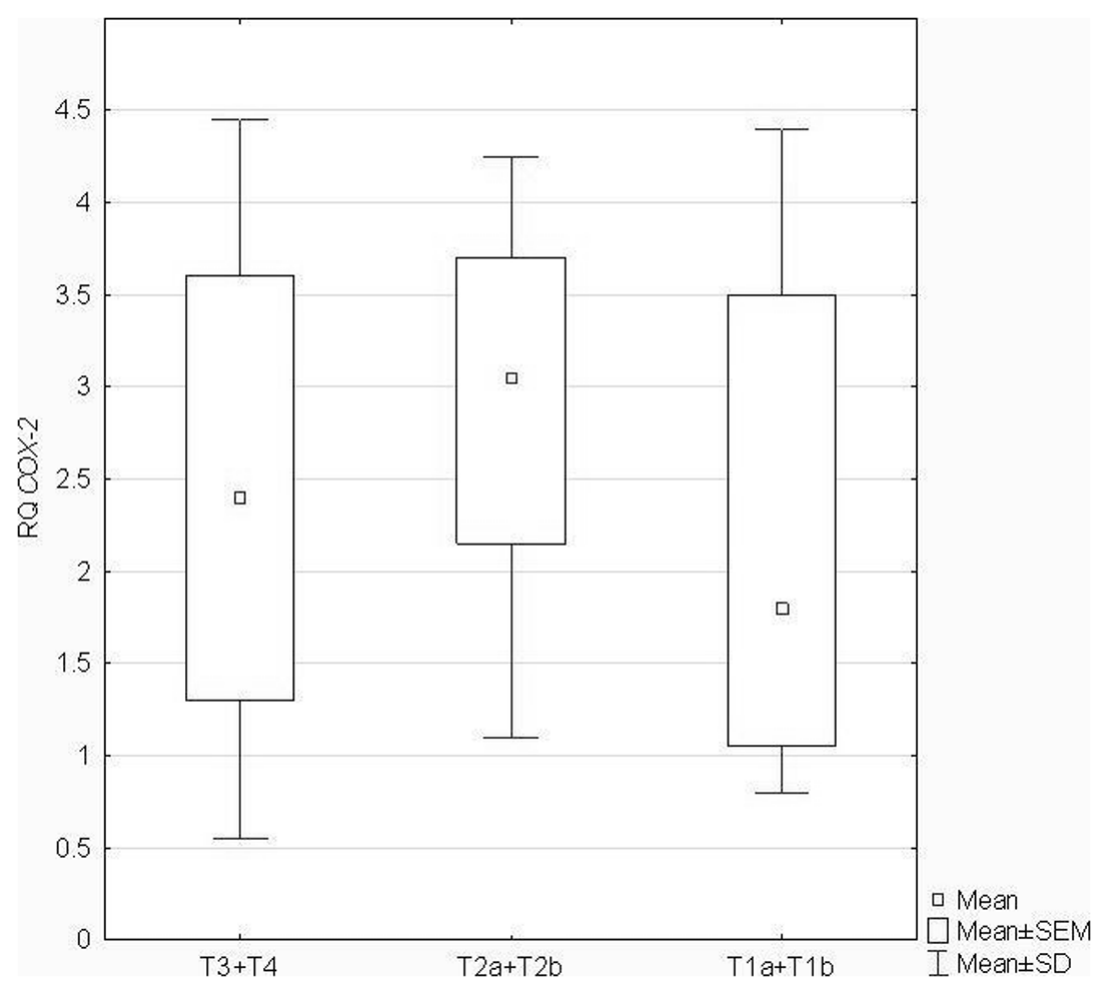
Box-and-whisker plots, representing the expression levels (mean RQ values) of *COX-2* gene in the studied tumor groups classified according to pTNM staging in relation to their size.

There was no statistically significant correlation between RQ values of *COX-2* and patients' age (P = 0.09), gender (P = 0.44) and history of smoking (P = 0.34) (ANOVA Kruskal-Wallis test, U Mann-Whitney's test followed by Spearman's rank correlation coefficient). Similarly, no statistically significant correlations were found between RQ values of *COX-2* and histopthological NSCLC subtypes: SCC *vs* NSCC (P = 0.51) and tumor AJCC classification (P = 0.28) (U Mann-Whitney's test). However, the association between tumor size according to pTNM staging and *COX-2* expression level was statistically significant (P = 0.041; ANOVA Kruskal–Wallis test), as presented in **Figure 2**. Neuman-Keuls' multiple comparison test revealed statistically significant differences in RQ values between T1a+T1b and T2a+T2b group (see **Table 5**).

**Table 5:** The results of Neuman-Keuls' multiple comparison test regarding differences in *COX-2* RQ values in the studied tumor groups classified according to pTNM staging in relation to the tumor size.

	T1a+t1b	T2a+T2b	T3+T4
**T1a+T1b**		**0.02459**	1000000
**T2a+T2b**	**0.02459**		0.27776
**T3+T4**	1.000000	0.27776	

**Figure 3:**
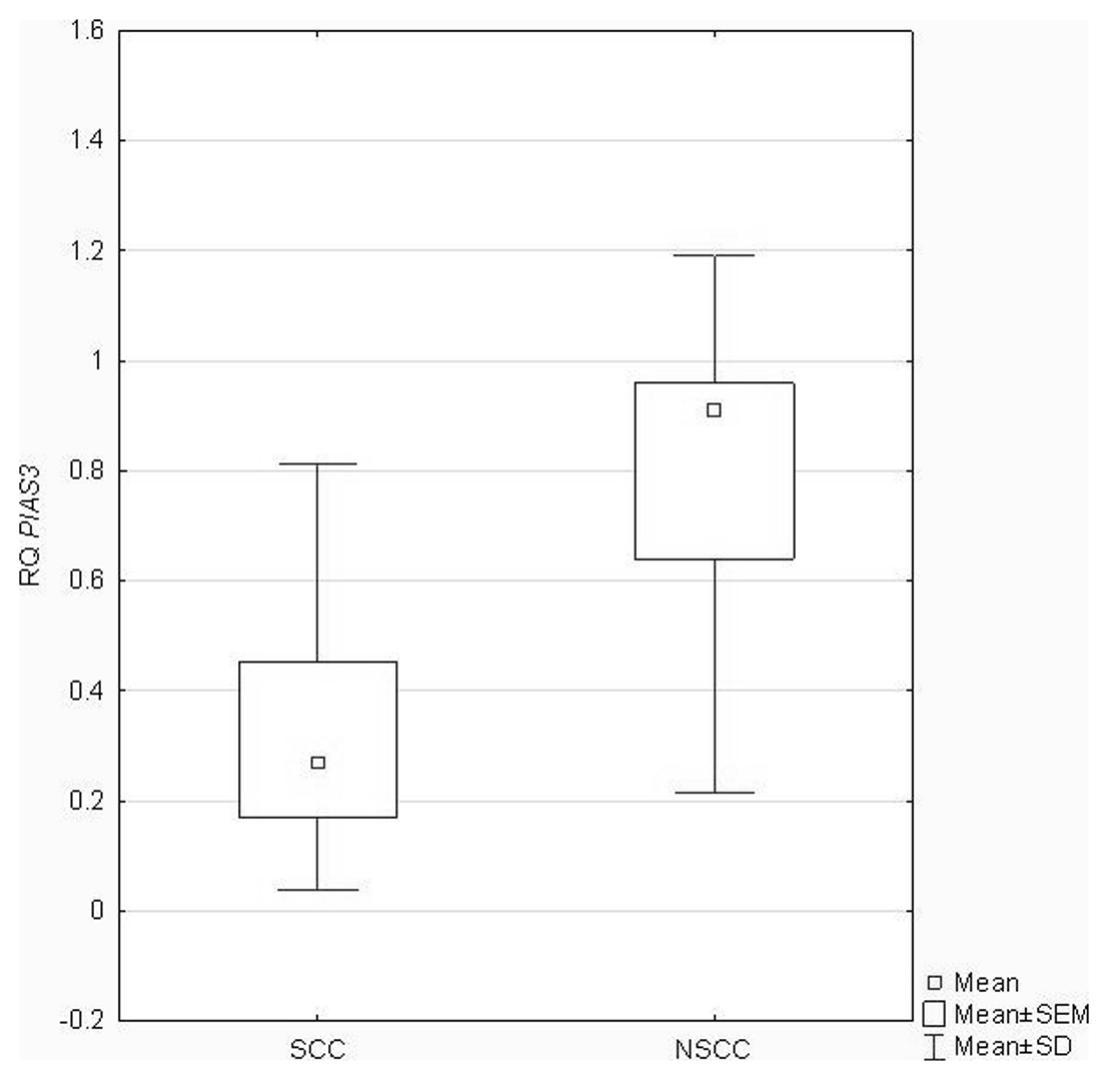
Box-and-whisker plots, representing *PIAS3* expression levels (mean RQ values) in the studied tumor groups classified according to the histopathological NSCLC subtypes.

Regarding *PIAS3* expression, statistical analysis did not reveal any significant correlations between the RQ values and patients' age (P = 0.48), gender (P = 0.37), and history of smoking (P = 0.58) (ANOVA Kruskal-Wallis test, U Mann-Whitney's test followed by Spearman's rank correlation coefficient), as well as tumor staging according to pTNM (P = 0.22) and AJCC classification (P = 0.12) (ANOVA Kruskal-Wallis test). Statistically significant differences were found between histopathological NSCLC subtypes (P = 0.017; U Mann-Whitney's test), as presented in **Figure 3**.

### 3. Statistical analysis of the reciprocal relationship between the expression of the studied genes

Finally, we assessed the reciprocal relationship between the expression levels of the studied genes. Spearman's rank correlation coefficient revealed statistically significant negative correlation between *STAT5B* and *PIAS3* genes (rho = −0.049, P = 0.04; Spearman's rank correlation) and positive correlation between *STAT5B* and *COX-2* genes (rho = 0.045, P = 0.027; Spearman's rank correlation). Additionally, statistically significant negative correlation between *STAT5B* and *PIAS3* genes was observed in T2a+T2b tumor group (rho = −0.43, P = 0.041; Spearman's rank correlation).

### 4. STAT5A and STAT5B immunoexpression levels

A quantitative measurements of STAT5A and STAT5B in NSCLC and control samples were performed using ELISA method. The mean values (ng/ml) obtained in the studied samples are presented in **Table 6**. Statistical analysis revealed that both STAT5A and STAT5B protein immunoexpression levels were significantly higher in lung cancer samples as compared with normal (macroscopically unchanged, control) lung tissues (P = 0.048 in case of STAT5A and P = 0.034 in case of STAT5B; U Mann-Whitney's test). However, the differences between NSCLC histopathological subtypes (SCC *vs* NSCC) weren't statistically significant, neither for STAT5B (P = 0.61; U Mann-Whitney's test) nor for STAT5A (P = 0.81; U Mann-Whitney's test).

**Table 6:** STAT5A and STAT5B protein levels (ng/ml) in the studied samples (controls, NSCLC and NSCLC histopathological subtypes).

Protein	NSCLC (n = 71)		Control (n = 20)		NSCLC subtypes			
	Mean [ng/ml]	Range [ng/ml]	Mean [ng/ml]	Range [ng/ml]	NSCC (n = 30)		SCC (n = 41)	
					Mean [ng/ml]	Range [ng/ml]	Mean [ng/ml]	Range [ng/ml]
**STAT5A**	16.73	0.39–19.25	4.88	0.33–10.22	16.73	0.39–19.25	15.62	0.39–18.71
	P = 0.048 U Mann-Whitney's test				P>0.05 U Mann-Whitney's test			
**STAT5B**	17.87	0.35–19.88	2.90	0.36–9.43	15.84	0.35–18.62	18.12	0.53–19.88
	P = 0.034 U Mann-Whitney's test				P>0.05 U Mann-Whitney's test			

**Figure 4:**
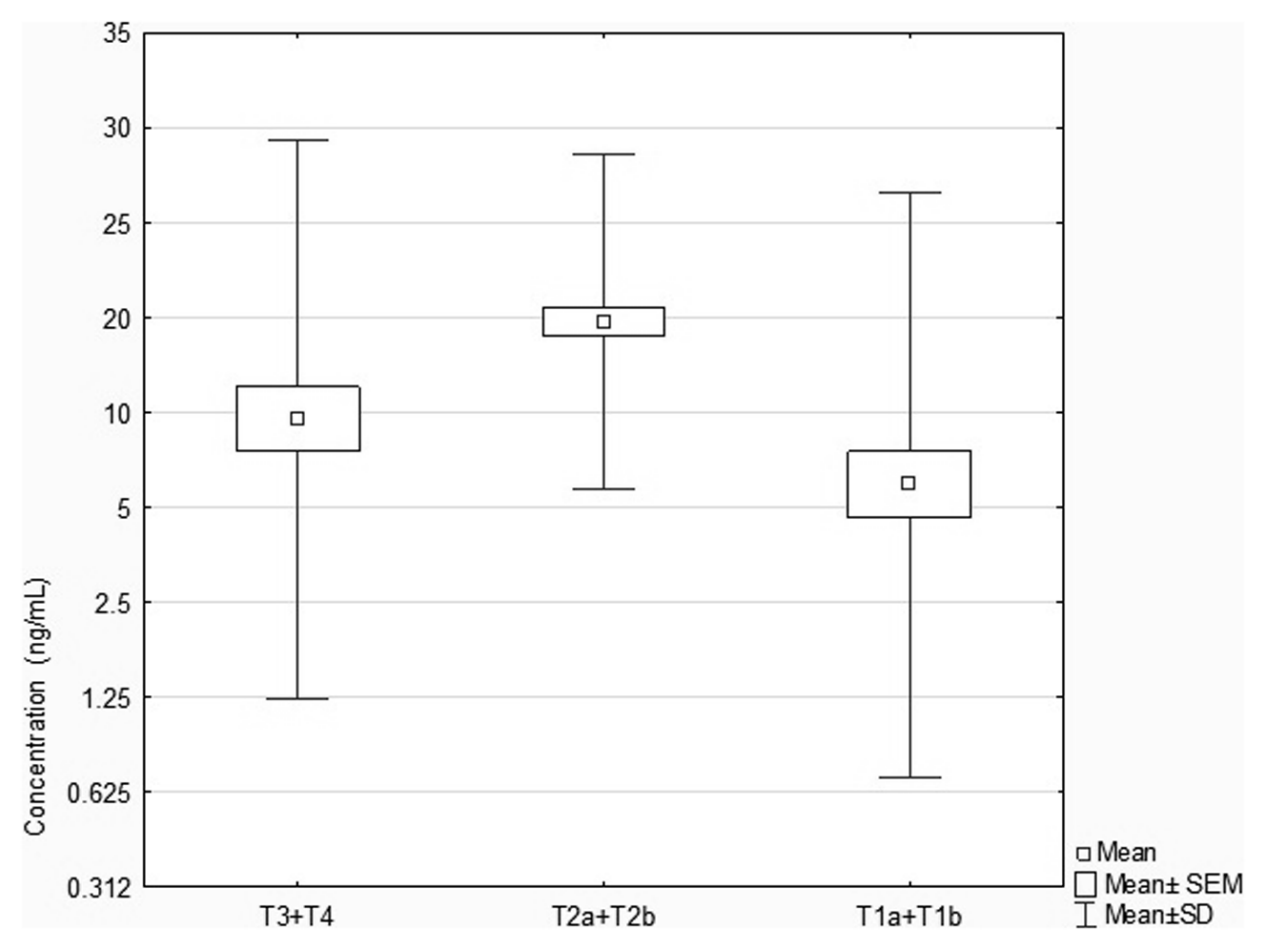
Box-and-whisker plots, representing STAT5B protein levels (ng/ml) in the studied tumor groups classified according to pTNM staging in relation to the tumor size.

STAT5B immunoexpression levels were significantly different between samples grouped according to pTNM classification in relation to tumor size (P = 0.045; ANOVA Kruskal-Wallis test), as presented in **Figure 4**. Neuman-Keuls' multiple comparison test revealed statistically significant differences between T1a+T1b and T2a+T2b group (see **Table 7**).

**Table 7:** The results of Neuman-Keuls' multiple comparison test regarding differences in STAT5B immunoexpression levels in the studied tumor groups classified according to pTNM staging in relation to the tumor size.

	T1a+T1b	T2a+T2b	T3+T4
**T1a+T1b**		**0.0046134**	0.92834
**T2a+T2b**	**0.0046134**		0.107350
**T3+T4**	0.92834	0.107350	

We didn't find any significant correlations between STAT5B immunoexpression level and patients' age (P = 0.61), gender (P = 0.40), the history of smoking assessed as PY (P = 0.63), and AJCC classification (P = 0.17) (ANOVA Kruskal-Wallis test, U Mann-Whitney's test followed by Spearman's rank correlation coefficient).

No significant correlations were found between pSTAT5A immunoexpression level and patients' age (P = 0.83), gender (P = 0.17), the history of smoking assessed as PY (P = 0.92), tumor size according to pTNM classification (P = 0.46), and AJCC classification (P = 0.98) (ANOVA Kruskal-Wallis test, U Mann-Whitney's test followed by Spearman's rank correlation coefficient).

### 5. Statistical analysis of the reciprocal relationship between *STAT5A/B* mRNA expression and STAT5A/B immunoexpression

**Figure 5:**
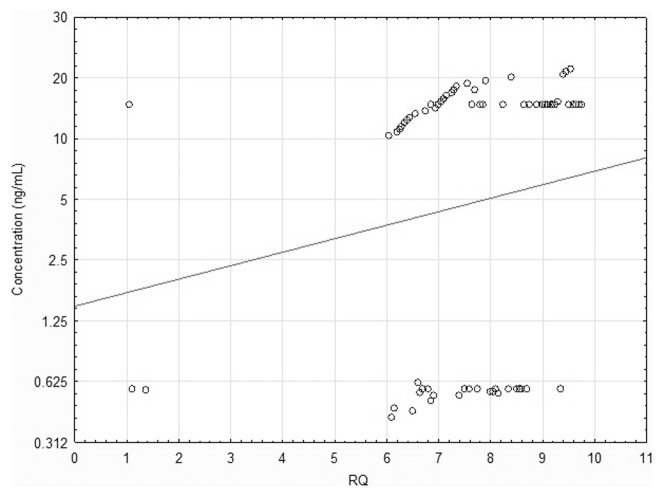
Positive correlation between STAT5B protein levels (ng/ml) and gene expression levels (RQ values) in NSCLC samples.

The positive correlation was found between the expression levels of *STAT5B* gene and the immunoexpression levels of STAT5B (rho = 0.755, P = 0.04; Spearman's rank correlation coefficient) in NSCLC samples, see **Figure 5**. No significant correlation was found between *STAT5A* gene expression and STAT5A protein level (P = 0.43, Spearman's rank correlation coefficient).

## Discussion

It is commonly known that deregulations of STAT signaling pathway can result in oncogenesis and the constitutive activation of *STAT5* is found in many human malignancies [10], [12], [24], including lung cancer [25]. Our study results confirmed the implication of *STAT5* isoforms in NSCLC development. We observed the increased expression levels of *STAT5* genes (A and B isoforms) in the majority of studied NSCLC tissues, both at mRNA and protein level. Our result are in accordance with observations of other investigators who have also reported STAT5A and STAT5B overexpression on mRNA and/or protein level in cancer tissue – prostate, breast, colorectal, esophageal and lung – when compared to normal tissue [10], [24]–[31].

Interestingly, it is known that STAT5A and STAT5B isoforms have distinct biological functions [32]–[33] and the expression patterns of these proteins differ among many types of cancers cells. Both STAT5A and STAT5B are key tumor growth stimulators, but STAT5B overexpression seems to be more significant in head and neck and prostate cancers, while STAT5A – in breast cancer [31], [34], [35]. However, the exact role of STAT5A as opposed to STAT5B in malignant cell transformation is not well understood. Recently, Tang et al. [36] have demonstrated differential regulation of mammary carcinoma cell behavior upon forced expression of STAT5A and STAT5B: STAT5B exhibited lower potency than STAT5A in enhancing survival and anchorage-independent growth of mammary carcinoma cells and exerted no effects on cell motility. In our study we assessed the immunoexpression of STAT5A and STAT5B proteins in lung cancer tissue and found it significantly higher than in normal lung tissue. The study performed by Sánchez-Ceja et al. [25] indicated the highest nuclear STAT5 expression in LCC (large cell carcinoma), lower in SCC and the lowest in AC (adenocarcinoma). In our study we included LCC and AC in one NSCC group due to the small number of LCC samples. However, we didn't find any significant differences between the groups. STAT5A and STAT5B protein levels were similar in the studied NSCLC subtypes, however, for procedural reasons, we could not analyze the location of proteins (nuclear or cytoplasmic).

The analysis of both gene isoforms on mRNA level in NSCLC samples revealed significantly higher expression of *STAT5B* isoform as compared with *STAT5A*. It might suppose different functions for both isoforms also in lung cancer cells, i.e., in result, different downstream target genes (including oncogenes) specifically induced by either STAT5A or STAT5B. The mechanism of lower *STAT5A* gene expression in NSCLC is not known, however in lymphomas *STAT5A* was found to be epigenetically silenced [37]. Consistent with the obtained results indicating high *STAT5B* expression in lung tumors was our analysis regarding STAT5B immunoexpression, as we observed positive correlation between these two parameters.

We documented also statistically significant differences in *STAT5B* expression levels depending on tumors size, i.e., significantly higher *STAT5B* expression in T2 group, which was additionally reflected by significantly higher STAT5B protein levels in this group. Thus, it could further suggest that STAT5B has more important implications than STAT5A in the pathogenesis of NSCLC. As so far there is no available published data concerning association of *STAT5B* expression with tumor progression and prognosis in NSCLC. The analysis of STAT5 immunohistochemical staining in NSCLC tissue, performed by Sánchez-Ceja et al. [25], suggested the association of STAT5 protein with advanced lung cancer stages. Regarding other tumor types the data are conflicting. The significant association of STAT5B protein expression with TNM stage was found in colorectal cancer [38], but not in gastric cancer [39]. Interesting results were obtained by Tang et al. [36], who proved a dual role of STAT5A in human mammary carcinoma cells: promotion of tumor formation by enhancing survival and anchorage-independent growth but simultaneous inhibition of tumor metastasis by suppression of cellular invasiveness.

In our study, we tried to establish – to our knowledge as a first research group – the relationship between *STAT5A* and *STAT5B* mRNA expression and patient's age, gender, and NSCLC histopathological subtype. Such an association could indicate an importance of *STAT5* mRNA expression level as a diagnostic tool in NSCLC. However, we didn't find any significant associations.

PIAS3 (protein inhibitor of activated STAT3), was originally identified as a specific inhibitor of the STAT3 signaling pathway [40]. Now it is well recognized that the PIAS family (PIAS1, PIAS3, PIASx and PIASy) regulates a variety of cytokine and transcription factors with downstream alterations in apoptosis, angiogenesis and a number of signaling pathways. Regarding PIAS3 and its role in lung tumorigenesis, it has been demonstrated that PIAS3 decreases lung cancer growth and increases the antitumor effects of EGFR inhibitors [20], activates the intrinsic apoptosis pathway *via* altered expression of Bcl-2 and Akt family members [21], [23], and interacts with several other transcription factors, including: ETS, EGR, NR1I2 and GATA1 [21], all of them playing important roles in cancer development. The STAT3 independent effect of PIAS3 includes also the repression of STAT5 transcriptional activity, as demonstrated by Rycyzyn and Clevenger [19]. In our study the expression level of *PIAS3* was negatively correlated with the expression level of *STAT5B* in the whole group of studied tumors, and especially, the negative correlation was observed in T2 tumor group.

In several cancers a decrease or loss of PIAS3 expression was demonstrated, indicating it as a protein with putative tumor suppressor function [22], but a paucity of studies were focused on lung cancer [20], [21], [23]. The results obtained in NSCLC cell lines indicated that overexpression of PIAS3 had growth inhibitory effect [20], [23]. Our analysis revealed the decreased *PIAS3* expression in the majority of the studied NSCLC samples. Moreover, it was significantly lower in NSCC as compared to SCC. Our results are similar to those obtained by Kluge et al. [20] who found *PIAS3* mRNA expression higher in SCC than in AC.

Cyclooxygenase (COX), is the key enzyme in the biosynthesis of the prostanoids and plays a central role in many important cellular processes, including inflammatory response, tumorigenesis, and tumor progression [41]. COX is comprised of three categories, including COX-1, COX-2 and COX-3 [15]. COX-2 converts arachidonic acid to bioactive lipids including prostaglandin E2 (PGE_2_), and their role in initiating and progressing tumors *in situ* was established. It was suggested that the overexpression of COX-2 and the resulting increase in PGE_2_ levels may represent a tumor strategy to escape immunosurveillance [42]. COX-2 overexpression was found by immunohistochemical method in well and moderately differentiated carcinomas of the lung, colon, and breast [18], [43]. In lung cancer, COX-2 overexpression was reported to inhibit apoptosis [16], promote angiogenesis [44] and metastasis [45]. In our study we confirmed increased expression of *COX-2* gene in the majority of NSCLC samples, regardless the histopathological subtype. In contrast, it was found that COX-2 protein expression in adenocarcinoma was significantly higher than that in squamous cell carcinoma [43], [46], [47]. These differences might be caused by further posttranslational modifications of COX-2 protein in lung cancer cells.

Interestingly, we observed statistically significant differences in *COX-2* expression with respect to tumor size – significantly higher gene expression levels were shown in T2a+T2b tumors as compared with T1a+T1b group. The meta-analysis combining 14 published studies, revealed a significant association between COX-2 expression and poor survival for stage I NSCLC (i.e., encompassing T1a, T1b and T2a tumors) [48]. However, the studies reporting the relationship between COX-2 expression and survival among lung cancer patients are inconsistent. The study performed by Groen et al. [49] confirmed that high *COX-2* expression predicted poor prognosis in NSCLC, but the results of more recent study have suggested that the prognostic role of COX-2 in NSCLC needs to be confirmed by further high-quality prospective studies [50].

In human lung adenocarcinoma cells it was found that COX-2 expression was stimulated *via* the activation of the STAT5 pathway thus showing that STAT5 plays a certain role, affecting the expression of COX-2 [14]. In our study the increased expression of *STAT5* was accompanied by increased expression of *COX-2* in nearly 70% of NSCLC samples. Moreover, expression levels of *STAT5B* and *COX-2* showed a significant positive correlation.

We didn't observe any statistically significant associations between expression values of the studied genes and clinical patients' characteristics: age, gender as well as tobacco addiction and consumption. Unfortunately, we can't refer our results to those obtained by others, because the available studies were carried out on lung cancer cell lines, rather than lung tissue derived from NSCLC patients.

## Conclusions

In our study we found significantly higher expression levels of *STAT5B* (both on mRNA and protein level) and *COX-2* gene in T2a+T2b as compared to T1a+T1b tumors. In the same tumor group, the negative correlation between *STAT5B* and *PIAS3* expression was statistically significant. It could indicate the role of *STAT5B* isoform and *COX-2* in lung cancer progression. In the absence of other reports on *STAT5* gene expression in lung cancer, the results require confirmation in a larger group of NSCLC patients. It seems, however, that the role of *STAT5B* in the development of lung cancer is more similar to that in prostate cancer than in breast cancer. Such knowledge is extremely important in the context of inhibition of *STAT5* in the potential treatment strategy of lung cancer. Additionally, the targeted therapy toward STAT activity might be based on the data regarding *PIAS3* and/or *COX-2* expression in lung cancer cells. Reassuming, our data confirmed the justification of ongoing clinical studies focused on selective inhibitors of COX and/or STAT in lung malignant cells.
